# Supplementary material for: Designing light-element materials with large effective spin-orbit coupling
Source: Nat Commun. 2022 Feb 17;13:919. doi: 10.1038/s41467-022-28534-y (PMC8854432; doi:10.1038/s41467-022-28534-y)
Supplement: Supplementary file 1 — Supplementary Information [file 41467_2022_28534_MOESM1_ESM.docx]

**Supplementary Information**

**Designing light-element materials with large effective spin-orbit coupling**

Jiayu Li^1^, Qiushi Yao^1^, Lin Wu^2^, Zongxiang Hu^1^, Boya Gao^1^, Xiangang Wan^2,*^ and Qihang Liu^1,3,4,*^

*^1^Shenzhen Institute for Quantum Science and Engineering (SIQSE) and Department of Physics, Southern University of Science and Technology, Shenzhen 518055, China*

*^2^National Laboratory of Solid State Microstructures and School of Physics, Nanjing University, Nanjing 210093, China and Collaborative Innovation Center of Advanced Microstructures, Nanjing University, Nanjing 210093, China*

*^3^Shenzhen Key Laboratory of Advanced Quantum Functional Materials and Devices, Southern University of Science and Technology, Shenzhen 518055, China*

*^4^Guangdong Provincial Key Laboratory for Computational Science and Material Design, Southern University of Science and Technology, Shenzhen 518055, China*

J. L., Q. Y., and L. W. contributed equally to this work.

^*^Emails: [xgwan@nju.edu.cn](mailto:xgwan@nju.edu.cn); [liuqh@sustech.edu.cn](mailto:liuqh@sustech.edu.cn)

Contents

[Supplementary Note 1. Mean-field approach on the interplay between correlation and spin-orbit coupling 3](#_Toc94029311)

[Supplementary Note 2. Space groups and layer groups that permit enhanced spin-orbit coupling effect and the corresponding Wyckoff Positions 5](#_Toc94029314)

[Supplementary Note 3. Collection of light-element transition-metal materials with potentially large SOC effect in C2DB database 13](#_Toc94029315)

[Supplementary Note 4. Results of monolayer hexagonal Fe_2_X_2_ (X = S, Se) 17](#_Toc94029316)

[Supplementary Note 5. Spin-orbit gap enhanced by general correlation 21](#_Toc94029319)

[Supplementary Note 6. Exchange coupling and magnetocrystalline anisotropy energy 25](#_Toc94029321)

# Supplementary Note 1. Mean-field approach on the interplay between correlation and spin-orbit coupling

## I. Orbital doublet case

In a lattice system, *d* orbital doublets permitted by the crystal field are $E_{1}=\left\{ d_{xz},d_{yz} \right\}$, $E_{2}=\left\{ d_{xy},d_{x^{2}-y^{2}} \right\}$, and $E_{3}=\left\{ d_{z^{2}},d_{x^{2}-y^{2}} \right\}$. The spin-orbit coupling (SOC) Hamiltonian reads

$$\hat{H}_{SOC}=\sum_{mm^{'}=1,\cdots,M,\sigma\sigma^{'}} \lambda\left\langle m,\sigma| \mathbf{L}\cdot\mathbf{S} | m^{'},\sigma^{'} \right\rangle\hat{C}_{m\sigma}^{\dagger}\hat{C}_{m^{'}\sigma^{'}}, (1)$$

where $\mathbf{L}$ and $\mathbf{S}$ are the angular momentum and spin operators; $\hat{C}_{m\sigma}^{\dagger}$ and $\hat{C}_{m\sigma}$ are the creation and annihilation operators on electron state at with orbital $m$, and spin $\sigma$, and $\lambda$ denotes the strength of the SOC effect. As we concentrate on the 3*d* systems with strong exchange interaction, we can only consider the spin-preserved term $\mathbf{L}\cdot\mathbf{S}\approx L_{z}S_{z}$. Taking the doublet $E_{1}$ as an example, we expand the SOC Hamiltonian matrix under the basis of $\left\{ \left. |2,1,\uparrow\right\rangle,\left. |2,-1,\uparrow\right\rangle,\left. |2,1,\downarrow\right\rangle,\left. |2,-1,\downarrow\right\rangle\right\}$ as

$$\tilde{H}_{SOC}=\frac{\lambda\hbar^{2}}{2}\left( \begin{matrix} 1 & & & \\ & -1 & & \\ & & -1 & \\ & & & 1 \end{matrix} \right), (2)$$

with $\left. |2,\pm1,\uparrow/\downarrow\right\rangle=-\left( \left. |d_{xz},\uparrow/\downarrow\right\rangle\pm i\left. |d_{yz},\uparrow/\downarrow\right\rangle\right)/\sqrt{2}$. Hence, the SOC Hamiltonian Eq. (S1) can now be expressed via new creation and annihilation operators as $\hat{H}_{SOC}=\frac{\lambda\hbar^{2}}{2}\left( \hat{n}_{1,\uparrow}-\hat{n}_{-1,\uparrow}-\hat{n}_{1,\downarrow}+\hat{n}_{-1,\downarrow} \right)$ with $\hat{n}_{m\sigma}=\hat{C}_{m\sigma}^{\dagger}\hat{C}_{m\sigma}$ the occupation operator. Since in the strong spin splitting condition, electron states near the Fermi level are dominated by single spin channel, where the effective Hamiltonian near the Fermi level is fully spin-polarized. In the following, we neglect the spin-down states and omitted the spin index in both the electron states and the creation/annihilation operators, *i.e.*, $\hat{H}_{SOC}=\frac{\lambda\hbar^{2}}{2}\left( \hat{n}_{1}-\hat{n}_{-1} \right)$.

Notice that the matrix representation of the projected orbital momentum is also diagonal in the basis of $\left. |2,\pm1 \right\rangle$, we express the SOC Hamiltonian in terms of orbital momentum operator as

$$\hat{H}_{SOC}=\frac{\lambda\hbar}{2}\hat{L}_{z}, (3)$$

where $\hat{L}_{z}=\sum_{m=\pm l_{z}} \left\langle m | L_{z} | m \right\rangle\hat{n}_{m}$ is the operator of the angular momentum. We should remind that the connection between angular momentum and SOC term is invalid for the doublet $E_{3}=\left\{ d_{z^{2}},d_{x^{2}-y^{2}} \right\}$ as $\left. |d_{z^{2}} \right\rangle=\left. |2,0 \right\rangle$ and $\left\langle d_{z^{2}},\sigma| \mathbf{L}\cdot\mathbf{S} | d_{x^{2}-y^{2}},\sigma^{'} \right\rangle=0$. The energy splitting of the degenerated orbitals $E_{1}$ due to the SOC effect is

$$\Delta E_{0}=\lambda\hbar^{2}. (4)$$

The magnitude of the splitting energy reveals the strength of the SOC effect.

Now we introduce the electron-electron correlation effect. As the case in 3*d* systems with significant exchange interaction but weak SOC, the effective on-site correlation effect is captured by the Coulomb repulsion between states with parallel spin as $\hat{H}_{c}=U_{\mathrm{eff}}\hat{n}_{1}\hat{n}_{-1}$, where $U_{\mathrm{eff}}=U-3J$ with $U$ and $J$ are the Hubbard and Hund parameters, respectively. In addition to the angular momentum operator $\hat{L}_{z}=\hbar\left( \hat{n}_{1}-\hat{n}_{-1} \right)$ as defined before, we also define the total occupation operator of the doublet as $\hat{n}=\hat{n}_{1}+\hat{n}_{-1}$. Hence, the correlation Hamiltonian is rewritten into

$$\hat{H}_{c}=\frac{U_{\mathrm{eff}}}{4}\left( \hat{n}^{2}-\frac{\hat{L}_{z}^{2}}{\hbar^{2}} \right). (5)$$

Under the Hartree-Fock approximation, we address $\hat{A}^{2}=\left( \bar{A}+\delta\hat{A} \right)^{2}\approx2\bar{A}\hat{A}-\bar{A}^{2}$ with the term $\left( \delta\hat{A} \right)^{2}$ neglected, where $\bar{A}$ denotes the expected value of $\hat{A}$. The correlation Hamiltonian becomes

$$\hat{H}_{c}\approx U_{\mathrm{eff}}\left[ \frac{\left| \bar{L}_{z} \right|}{2\hbar^{2}}\hat{L}_{z}+\hat{\xi}\left( \bar{n},\bar{L}_{z} \right) \right], (6)$$

where we defined the energy shift

$$\hat{\xi}\left( \bar{n},\bar{L}_{z} \right)=\frac{1}{2}\left[ \bar{n}\left( \hat{n}-\frac{\bar{n}}{2} \right)+\frac{\bar{L}_{z}^{2}}{2\hbar^{2}} \right]. (7)$$

Recalling that the SOC Hamiltonian takes the form of $\hat{H}_{SOC}=\lambda\hbar\hat{L}_{z}/2$ in Eq. (3), we find that the joint effect of SOC and correlation leads to an effective Hamiltonian of

$$\hat{H}_{c}+\hat{H}_{SOC}\approx\frac{\lambda_{\mathrm{eff}}\hbar}{2}\hat{L}_{z}+U_{\mathrm{eff}}\hat{\xi}\left( \bar{n},\bar{L}_{z} \right), (8)$$

which produces a modified SOC effect with an effective SOC parameter

$$\lambda_{\mathrm{eff}}=\lambda+U_{\mathrm{eff}}\frac{\left| \bar{L}_{z} \right|}{\hbar^{3}}. (9)$$

Hence, the energy splitting induced by the SOC effect is now enhanced as

$$\Delta E_{\mathrm{eff}}=\Delta E_{0}+U_{\mathrm{eff}}\frac{\left| \bar{L}_{z} \right|}{\hbar}, (10)$$

where $\Delta E_{0}$ is the unmodified splitting energy Eq. (4). As the splitting energy reveals the strength of the SOC effect, we find that the correlation effect considered in Eq. (5) can dramatically enlarge the SOC effect. $\bar{L}_{z}$ is also a function of $\lambda_{\mathrm{eff}}$ or $U_{\mathrm{eff}}$, and depends on the inter-site hopping term. As the intrinsic SOC is relatively small, $\left| \bar{L}_{z} \right|$ can be expressed as a linear function of $\lambda$: $\left| \bar{L}_{z} \right|=\chi\lambda$, where $\chi$ is the susceptibility depending on lattice and hopping term. Given a small intrinsic $\lambda$ and $\left| \bar{L}_{z} \right|=\chi\lambda$, Eq. (9) outputs a modified $\lambda^{'}$ through Eq. (9) and a corresponding $\left| \bar{L}_{z}^{'} \right|=\chi\lambda^{'}$. The self-consistent condition is reached once the input and output are identical, *i.e.*, $\lambda_{\mathrm{eff}}=\lambda+U_{\mathrm{eff}}\frac{\chi\lambda_{\mathrm{eff}}}{\hbar^{3}}$. Hence the self-consistent solution of the effective SOC gap $\Delta E_{\mathrm{eff}}=\lambda_{\mathrm{eff}}\hbar^{2}$ solves

$$\frac{\Delta E_{eff}}{\Delta E_{0}}\approx\left[ 1-\frac{\left( U-3J \right)\chi}{\hbar^{3}} \right]^{-1}. (11)$$

For the orbital doublet $E_{2}$, the derivation and results are similar.

## II. Orbital triplet case

In a periodic crystal, there is only one *d* orbital triplet allowed by the local crystal field as $T=\left\{ d_{xy},d_{yz},d_{xz} \right\}$. The SOC matrix in the basis $\left\{ \left. |1 \right\rangle,\left. |-1 \right\rangle,\left. |0 \right\rangle\right\}$ reads

$$\tilde{H}_{SOC}=\frac{\lambda\hbar^{2}}{2}\left( \begin{matrix} 1 & & \\ & -1 & \\ & & 0 \end{matrix} \right), (12)$$

where $\left. |\pm1 \right\rangle\equiv\left( \left. |d_{xz},\uparrow\right\rangle\pm i\left. |d_{yz},\uparrow\right\rangle\right)/\sqrt{2}$ and $\left. |0 \right\rangle\equiv\left. |d_{xy},\uparrow\right\rangle$. Under the same manner, we find that the SOC Hamiltonian recovers the form as in Eq (3) as the state $\left. |0 \right\rangle$ has no projected angular momentum. The difference is revealed in the correlation term as $\hat{H}_{c}=U_{\mathrm{eff}}\left( \hat{n}_{1}\hat{n}_{0}+\hat{n}_{-1}\hat{n}_{0}+\hat{n}_{1}\hat{n}_{-1} \right)$. With the definitions of the total occupation operator $\hat{n}$, we rewrite $\hat{H}_{c}$ as

$$\hat{H}_{c}=\frac{U_{\mathrm{eff}}}{4}\left( \hat{n}^{2}-\frac{\hat{L}_{z}^{2}}{\hbar^{2}} \right)+\frac{U_{\mathrm{eff}}}{2} \left( \hat{n}-\frac{3}{2} \right)\hat{n}_{0}. (13)$$

Notice that the first term above is same as Eq. (5), which leads to the same mean-field result of Eq. (6). For the second term containing $\hat{n}$ and $\hat{n}_{0}$, it can be absorbed into the energy shifting term $\hat{\xi}\left( \bar{n},\bar{L}_{z},\bar{n}_{0} \right)$ due to its independence on $\hat{L}_{z}$.

Comparing orbital doublet in Eq. (5) and triplet in Eq. (13), we note the term $-\hat{L}_{z}^{2}$ is essential for the correlation-enhanced SOC effect as it provides an energy cost on state of electrons with opposite angular momenta. Then the splitting between single-particle energies of different orbital angular momenta is enlarged. Therefore, the effective SOC is also enhanced in orbital triplet case.

# Supplementary Note 2. Space groups and layer groups that permit enhanced spin-orbit coupling effect and the corresponding Wyckoff Positions

For a single ion, the quantum states of the spin-polarized *d*-shell electrons are five-fold degenerated due to the spherical symmetry. Exposed to a lattice environment, however, this high degeneracy is no longer preserved as the symmetry broken down from continuous to discrete. According to the group theory, the high-dimensional irreducible representation (irrep) corresponds to the degenerated states. The five-dimensional irrep $A^{d}$ of the 3D rotation group $SO(3)$ needs to be decomposed into direct sum of the irreps of the site-symmetry groups of Wyckoff positions in space groups. Considering the orbital multiplets permitted in 32 point groups, we simply find that there are four decompositions corresponding to four classes: I. tetragonal $A^{d}=A_{1}\oplus B_{1}\oplus B_{2}\oplus E$; II. triagonal $A^{d}=A_{1}\oplus2E$; III. hexagonal $A^{d}=A_{1}\oplus E_{1}\oplus E_{2}$, and IV. cubic $A^{d}=E\oplus T$, with $A_{i},B_{i}$ the 1D irreps, $E_{i}$ the 2D irreps, and $T$ the 3D irreps. Point groups with one of the four decompositions are listed in Supplementary Table 1.

After analyzing all the space groups, we observe 125 space groups out of 230, inside which the site-symmetry groups of the Wyckoff positions are those listed in Table S1. We present those 125 space groups along with the Wyckoff positions and the corresponding site-symmetry groups in Supplementary Table 2. One can directly predict that a material with 3*d* elements may have a greatly enhanced SOC effect only if the Wyckoff positions of the 3*d* atoms are given in Supplementary Table 2.

As the study of 2D materials are of great interest recently, we reproduce the above analysis on 80 layer groups analogously, where we extract 32 layer groups out of 80. In Supplementary Table 3, we deliver those 32 layer groups with the Wyckoff positions and the corresponding site-symmetry groups. In a same manner, one can predict that a 2D materials with 3*d* elements could possess an enhanced SOC effect once the Wyckoff positions of the 3*d* elements are listed in Supplementary Table 3.

**Supplementary Table 1.** **24 Single point groups with orbital multiplet for *d* orbits.**

| **Class** | **Decomposition (Basis)** | **Point groups** |
| --- | --- | --- |
| Tetragonal | $A^{d}=A_{1}\oplus B_{1}\oplus B_{2}\oplus E$  $A_{1}:d_{3z^{2}-r^{2}},B_{1}:d_{x^{2}-y^{2}}$  $B_{2}:d_{xy},E:\left\{ d_{xz},d_{yz} \right\}$ | C_4_(4), S_4_(-4), C_4h_(4/m), D_4_(422),  C_4v_(4mm), D_2d_(-42m), D_4h_(4/mmm) |
| Triagonal | $A^{d}=A_{1}\oplus2E$  $A_{1}:d_{3z^{2}-r^{2}}$  $E:\left\{ d_{x^{2}-y^{2}},d_{xy} \right\};\left\{ d_{xz},d_{yz} \right\}$ | C_3_(3), S_6_(-3), D_3_(32),  C_3v_(3m), D_3d_(-3m) |
| Hexagonal | $A^{d}=A_{1}\oplus E_{1}\oplus E_{2}$  $A_{1}:d_{3z^{2}-r^{2}}$  $E_{1}:\left\{ d_{x^{2}-y^{2}},d_{xy} \right\},E_{2}:\left\{ d_{xz},d_{yz} \right\}$ | C_6_(6), C_3h_(-6), C_6h_(6/m), D_6_(622),  C_6v_(6mm), D_3h_(-62m), D_6h_(6/mmm) |
| Cubic | $A^{d}=E\oplus T$  $E:\left\{ d_{x^{2}-y^{2}},d_{3z^{2}-r^{2}} \right\}$  $T:\left\{ d_{xz},d_{yz},d_{xy} \right\}$ | T(23), T_h_(m-3), O(432),  T_d_(-43m), O_h_(m-3m) |

**Supplementary Table 2.** **All the 125 space groups in which the site-symmetry groups of Wyckoff positions permit orbital multiplets.** (SG: Space Group)

| **Space group** | **Wyckoff position** | **Site-symmetry group** | **Space group** | **Wyckoff position** | **Site-symmetry group** |
| --- | --- | --- | --- | --- | --- |
| P4 (SG75) | 1a,1b | 4 | P-62m (SG189) | 4h | 3 |
| I4 (SG79) | 2a | 4 |  | 2e | 3m |
| P-4 (SG81) | 1a,1b,1c,1d | -4 |  | 2c,2d | -6 |
| I-4 (SG82) | 2a,2b,2c,2d | -4 |  | 1a,1b | -62m |
| P4/m (SG83) | 2g,2h | 4 | P-62c (SG190) | 4e,4f | 3 |
|  | 1a,1b,1c,1d | 4/m |  | 2b,2c,2d | -6 |
| P4_2_/m (SG84) | 2e,2f | -4 |  | 2a | 32 |
| P4/n (SG85) | 2c | 4 | P6/mmm (SG191) | 4h | 3m |
|  | 2a,2b | -4 |  | 2e | 6mm |
| P4_2_/n (SG86) | 2a,2b | -4 |  | 2c,2d | -62m |
| I4/m (SG87) | 4e | 4 |  | 1a,1b | 6/mmm |
|  | 4d | -4 | P6/mcc (SG192) | 8h | 3 |
|  | 2a,2b | 4/m |  | 4e | 6 |
| I4_1_/a (SG88) | 4a,4b | -4 |  | 4d | -6 |
| P422 (SG89) | 2g2h | 4 |  | 4c | 32 |
|  | 1a,1b,1c,1d | 422 |  | 2b | 6/m |
| P42_1_2 (SG90) | 2c | 4 |  | 2a | 622 |
| I422 (SG97) | 4e | 4 | P6_3_/mcm (SG193) | 8h | 3 |
|  | 2a,2b | 422 |  | 4e | 3m |
| P4mm (SG99) | 1a,1b | 4mm |  | 4d | 32 |
| P4bm (SG100) | 2a | 4 |  | 4c | -6 |
| P4cc (SG103) | 2a,2b | 4 |  | 2b | -3m |
| P4nc (SG104) | 2a | 4 |  | 2a | -62m |
| I4mm (SG107) | 2a | 4mm | P6_3_/mmc (SG194) | 4e,4f | 3m |
| I4cm (SG108) | 4a | 4 |  | 2b,2c,2d | -62m |
| P-42m (SG111) | 1a,1b,1c,1d | -42m |  | 2a | -3m |
| P-42c (SG112) | 2e,2f | -4 | P23 (SG195) | 4e | 3 |
| P-42_1_m (SG113) | 2a,2b | -4 |  | 1a,1b | 23 |
| P-42_1_c (SG114) | 2a,2b | -4 | F23 (SG196) | 16e | 3 |
| P-4m2 (SG115) | 1a,1b,1c,1d | -42m |  | 4a,4b,4c,4d | 23 |
| P-4c2 (SG116) | 2c,2d | -4 | I23 (SG197) | 8c | 3 |
| P-4b2 (SG117) | 2a,2b | -4 |  | 2a | 23 |
| P-4n2 (SG118) | 2a,2b | -4 | P2_1_3 (SG198) | 4a | 3 |
| I-4m2 (SG119) | 2a,2b,2c,2d | -42m | I2_1_3 (SG199) | 8a | 3 |
| I-4c2 (SG120) | 2b,2c | -4 | Pm-3 (SG200) | 8i | 3 |
| I-42m (SG121) | 4d | -4 |  | 1a,1b | m-3 |
|  | 2a,2b | -42m | Pn-3 (SG201) | 8e | 3 |
| I-42d (SG122) | 4a,4b | -4 |  | 4b,4c | -3 |
| P4/mmm (SG123) | 2g,2h | 4mm |  | 2a | 23 |
|  | 1a,1b,1c,1d | 4/mmm | Fm-3 (SG202) | 32f | 3 |
| P4/mcc (SG124) | 4g,4h | 4 |  | 8c | 23 |
|  | 2b,2d | 4/m |  | 4a,4b | m-3 |
|  | 2a,2c | 422 | Fd-3 (SG203) | 32e | 3 |
| P4/nbm (SG125) | 4g | 4 |  | 16c,16d | -3 |
|  | 2c,2d | -42m |  | 8a,8b | 23 |
|  | 2a,2b | 422 | Im-3 (SG204) | 16f | 3 |
| P4/nnc (SG126) | 4e | 4 |  | 8c | -3 |
|  | 4d | -4 |  | 2a | m-3 |
|  | 2a,2b | 422 | Pa-3 (SG205) | 8c | 3 |
| P4/mbm (SG127) | 4e | 4 |  | 4a,4b | -3 |
|  | 2a,2b | 4/m | Ia-3 (SG206) | 16c | 3 |
| P4/mnc (SG128) | 4e | 4 |  | 8a,8b | -3 |
|  | 2a,2b | 4/m | P432 (SG207) | 8g | 3 |
| P4/nmm (SG129) | 2c | 4mm |  | 6e,6f | 4 |
|  | 2a,2b | -42m |  | 3c,3d | 422 |
| P4/ncc (SG130) | 4c | 4 |  | 1a,1b | 432 |
|  | 4b | -4 | P4_2_32 (SG208) | 8g | 3 |
| P4_2_/mmc (SG131) | 2e,2f | -42m |  | 4b,4c | 32 |
| P4_2_/mcm (SG132) | 2b,2d | -42m |  | 2a | 23 |
| P4_2_/nbc (SG133) | 4d | -4 | F432 (SG209) | 32f | 3 |
| P4_2_/nnm (SG134) | 2a,2b | -42m |  | 24e | 4 |
| P4_2_/mbc (SG135) | 4b | -4 |  | 8c | 23 |
| P4_2_/mnm (SG136) | 4d | -4 |  | 4a,4b | 432 |
| P4_2_/nmc (SG137) | 2a,2b | -42m | F4_1_32 (SG210) | e | 3 |
| P4_2_/ncm (SG138) | 4b | -4 |  | 16c,16e | 32 |
| I4/mmm (SG139) | 4e | 4mm |  | 8a,8b | 23 |
|  | 4d | -42m | I432 (SG211) | 16f | 3 |
|  | 2a,2b | 4/mmm |  | 12e | 4 |
| I4/mcm (SG140) | 8f | 4 |  | 8c | 32 |
|  | 4c | 4/m |  | 6b | 422 |
|  | 4b | -42m |  | 2a | 432 |
|  | 4a | 422 | P4_3_32 (SG212) | 8c | 3 |
| I41/amd (SG141) | 4a,4b | -42m |  | 4a,4b | 32 |
| I4_1_/acd (SG142) | 8a | -4 | P4_1_32 (SG213) | 8c | 3 |
| P3 (SG143) | 1a,1b,1c | 3 |  | 4a,4b | 32 |
| R3 (SG146) | 3a | 3 | I4132 (SG214) | 16e | 3 |
| P-3 (SG147) | 2c,2d | 3 |  | 8a,8b | 32 |
|  | 1a,1b | -3 | P-43m (SG215) | 4e | 3m |
| R-3 (SG148) | 6c | 3 |  | 3c,3d | -42m |
|  | 3a,3b | -3 |  | 1a,1b | -43m |
| P312 (SG149) | 2g,2h,2i | 3 | F-43m (SG216) | 16e | 3m |
|  | 1a,1b,1c,1d,  1e,1f | 32 |  | 4a,4b,4c,4d | -43m |
| P321 (SG150) | 2c,2d | 3 | I-43m (SG217) | 12d | -4 |
|  | 1a,1b | 32 |  | 8c | 3m |
| R32 (SG155) | 6c | 3 |  | 6b | -42m |
|  | 3a,3b | 32 |  | 2a | -43m |
| P3m1 (SG156) | 1a,1b,1c | 3m | P-43n (SG218) | 8e | 3 |
| P31m (SG157) | 2b | 3 |  | 6c,6d | -4 |
|  | 1a | 3m |  | 2a | 23 |
| P3c1 (SG158) | 2a,2b,2c | 3 | F-43c (SG219) | 32e | 3 |
| P31c (SG159) | 2a,2b | 3 |  | 24c,24d | -4 |
| R3m (SG160) | 3a | 3m |  | 8a,8b | 23 |
| R3c (SG161) | 6a | 3 | I-43d (SG220) | 16c | 3 |
| P-31m (SG162) | 4h | 3 |  | 12a,12b | -4 |
|  | 2e | 3m | Pm-3m (SG221) | 8g | 3m |
|  | 2c,2d | 32 |  | 6e,6f | 4mm |
|  | 1a,1b | -3m |  | 3c,3d | 4/mmm |
| P-31c (SG163) | 4e,4f | 3 |  | 1a,1b | m-3m |
|  | 2a,2c,2d | 32 | Pn-3n (SG222) | 16f | 3 |
|  | 2b | -3 |  | 12e | 4 |
| P-3m1 (SG164) | 2c,2d | 3m |  | 12d | -4 |
|  | 1a,1b | -3m |  | 8c | -3 |
| P-3c1 (SG165) | 4c,4d | 3 |  | 6b | 422 |
|  | 2b | -3 |  | 2a | 432 |
|  | 2a | 32 | Pm-3n (SG223) | 16i | 3 |
| R-3m (SG166) | 6c | 3m |  | 8e | 32 |
|  | 3a,3b | -3m |  | 6c,6d | -42m |
| R-3c (SG167) | 12c | 3 |  | 2a | m-3 |
|  | 6b | -3 | Pn-3m (SG224) | 8e | 3m |
|  | 6a | 32 |  | 6d | -42m |
| P6 (SG168) | 2b | 3 |  | 4b,4c | -3m |
|  | 1a | 6 |  | 2a | -43m |
| P6_3_ (SG173) | 2a,2b | 3 | Fm-3m (SG225) | 32f | 3m |
| P-6 (SG174) | 2g,2h,2i | 3 |  | 24e | 4mm |
|  | 1a,1b,1c,1d,  1e,1f | -6 |  | 8c | -43m |
| P6/m (SG175) | 4h | 3 |  | 4a,4b | m-3m |
|  | 2e | 6 | Fm-3c (SG226) | 643 | 3 |
|  | 2c,2d | -6 |  | 48f | 4 |
|  | 1a,1b | 6/m |  | 24d | 4/m |
| P6_3_/m (SG176) | 4e,4f | 3 |  | 24c | -42m |
|  | 2a,2c,2d | -6 |  | 8b | m-3 |
|  | 2b | -3 |  | 8a | 432 |
| P622 (SG177) | 4h | 3 | Fd-3m (SG227) | 32e | 3m |
|  | 2e | 6 |  | 16c,16d | -3m |
|  | 2c,2d | 32 |  | 8a,8b | -43m |
|  | 1a,1b | 622 | Fd-3c (SG228) | 64e | 3 |
| P6_3_22 (SG182) | 4e,4f | 3 |  | 48d | -4 |
|  | 2a,2b,2c,2d | 32 |  | 32c | -3 |
| P6mm (SG183) | 2b | 3m |  | 32b | 32 |
|  | 1a | 6mm |  | 2a | 23 |
| P6cc (SG184) | 4b | 3 | Im-3m (SG229) | 16f | 3m |
|  | 2a | 6 |  | 12e | 4mm |
| P6_3_cm (SG185) | 4b | 3 |  | 12d | -42m |
|  | 2a | 3m |  | 8c | -3m |
| P6_3_mc (SG186) | 2a,2b | 3m |  | 6b | 4/mmm |
| P-6m2 (SG187) | 2g,2h,2i | 3m |  | 2a | m-3m |
|  | 1a,1b,1c,1d,  1e,1f | -62m | Ia-3d (SG230) | 32e | 3 |
| P-6c2 (SG188) | 4g,4h,4i | 3 |  | 24d | -4 |
|  | 2b,2d,2f | -6 |  | 16b | 32 |
|  | 2a,2c,2e | 32 |  | 16a | -3 |

**Supplementary Table 3.** **All the 32 layer groups in which the site-symmetry groups of Wyckoff positions permit orbital multiplets.** (LG: Layer Group)

| **Layer group** | **Wyckoff position** | **Site-symmetry group** | **Layer group** | **Wyckoff position** | **Site-symmetry group** |
| --- | --- | --- | --- | --- | --- |
| p4 (LG49) | 1a,1b | 4 | p31m (LG70) | 2b | 3 |
| p-4 (LG50) | 1a,1b | -4 |  | 1a | 3m |
| p4/m (LG51) | 1a,1b | 4/m | p-31m (LG71) | 4e | 3 |
| p4/n (LG52) | 2b | 4 |  | 2c | 3m |
|  | 2a | -4 |  | 2b | 32 |
| p422 (LG53) | 2d,2e | 4 |  | 1a | -3m |
|  | 1a,1b | 422 | p-3m1 (LG72) | 2b,2c | 3m |
| p42_1_2 (LG54) | 2b | 4 |  | 1a | -3m |
| p4mm (LG55) | 1a,1b | 4mm | p6 (LG73) | 2b | 3 |
| p4bm (LG56) | 2a | 4 |  | 1a | 6 |
| p-42m (LG57) | 1a,1b | -42m | p-6 (LG74) | 2d,2e,2f | 3 |
| p-42_1_m (LG58) | 2a | -4 |  | 1a,1b,1c | -6 |
| p-4m2 (LG59) | 1a,1b | -42m | p6/m (LG75) | 4e | 3 |
| p-4b2 (LG60) | 2a | -4 |  | 2c | 6 |
| p4/mmm (LG61) | 2d,2e | 4mm |  | 2b | -6 |
|  | 1a,1b | 4/mmm |  | 1a | 6/m |
| p4/nbm (LG62) | 4d | 4 | p622 (LG76) | 4e | 3 |
|  | 2b | -42m |  | 2c | 6 |
|  | 2a | 422 |  | 2b | 32 |
| p4/mbm (LG63) | 4c | 4 |  | 1a | 622 |
|  | 2a | 4/m | p6mm (LG77) | 2b | 3m |
| p4/nmm (LG64) | 2b | 4mm |  | 1a | 6mm |
|  | 2a | -42m | p-6m2 (LG78) | 2d,2e,2f | 3m |
| p3 (LG65) | 1a,1b,1c | 3 |  | 1a,1b,1c | -6m2 |
| p-3 (LG66) | 2b,2c | 3 | p-62m (LG79) | 4e | 3 |
|  | 1a | -3 |  | 2c | 3m |
| p312 (LG67) | 2d,2e,2f | 3 |  | 2b | -6 |
|  | 1a,1b,1c | 32 |  | 1a | -62m |
| p321 (LG68) | 2b,2c | 3 | p6mmm (LG80) | 4e | 3m |
|  | 1a | 32 |  | 2c | 6mm |
| p3m1 (LG69) | 1a,1b,1c | 3m |  | 2b | -6m2 |
|  |  |  |  | 1a | 6/mmm |

# Supplementary Note 3. Collection of light-element transition-metal materials with potentially large SOC effect in C2DB database

As the Computational 2D materials database (C2DB)^1, 2^ contains a wealth of computed properties for plenty of 2D materials with over 300 citations, we apply the C2DB as our material pool, in which organizes more than 1600 2D materials. First, we select 859 2D materials containing 3*d*/4*d* transition metal atoms inside the element set Sc~Ni for 3*d* and Y~Rh. Among them, we concentrate on 560 materials, of which the layer groups belong to the ones mentioned in Supplementary Table 3, and so do the Wyckoff positions of the transition metal elements. Then we further perform the routine SOC-free DFT calculation without considering the correlation effect (set U=0) on 542 materials to check out the energy band near the Fermi energy. In this step, a candidate is picked once the orbital-doublet bands of the transition metal elements are partially occupied, where the degenerated states at certain momentum are located within 1 eV around the Fermi energy. Finally, we pin down 71 candidates to most potentially generate the correlation-enhanced SOC effect while further considering the correlation effect with finite U, where the full list is presented in Supplementary Table 4 and their structures are provided at [<https://github.com/jylicmp/Structures>].

After obtaining the candidates, we perform elaborate DFT calculation on them with the consideration of the SOC and correlation effect. The results of the SOC gap among the candidates show good consistency with our theorical prediction, in which the SOC gap is typically valued at hundreds of meV, i.e., an order larger than the general strength of the on-site SOC effect in 3*d* series. We will present the detail results in **Supplementary** **Note 4** via two representative compounds Fe_2_S_2_ and Fe_2_Se_2_. With the SOC gap opened, the band topology of the occupied states can be directly determined by checking the Chern number or the chiral edge modes. Within the construction of the tight-binding model in Wannier-representation, we obtain 9 quantum anomalous Hall (QAH) insulators among 71 candidates with finite Chern numbers, which are denoted as “QAH” in Supplementary Table 4. As the SOC gap is enhanced by the correlation effect, the nontrivial gaps of the QAH insulators are naturally enlarged to resist the thermal fluctuation. As a simple application of our theory, it is an efficient way to design large amounts of high-temperature QAH insulators instead of case-by-case study.

**Supplementary Table 4.** **71 2D candidate materials that can produce the correlation-enhanced SOC effect screened from the Computational 2D materials database^1, 2^.** In the table, LG denotes layer group and TME@WPs denotes the Wyckoff positions that the transition metal elements locate at. Tags “QAH” in the “Notes” column label the materials as the candidates of high-temperature QAH insulators.

| **Compounds** | **LG** | **TME@WPs** | **Notes** |
| --- | --- | --- | --- |
| CrS2_id1451 | p-4m2 (LG59) | Cr@1a |  |
| CrSe2_id443 | p-4m2 (LG59) | Cr@1a |  |
| FeCl2_id1463 | p-4m2 (LG59) | Fe@1a |  |
| FeBr2_id453 | p-4m2 (LG59) | Fe@1a |  |
| AlNi5Cl2_id727 | p4/mmm (LG61) | Ni@1a |  |
| AlNi5Br2_id1736 | p4/mmm (LG61) | Ni@1a |  |
| GaNi5Cl2_id1739 | p4/mmm (LG61) | Ni@1a |  |
| GaNi5Br2_id2751 | p4/mmm (LG61) | Ni@1a |  |
| InNi5Cl2_id2754 | p4/mmm (LG61) | Ni@1a |  |
| InNi5Br2_id3770 | p4/mmm (LG61) | Ni@1a |  |
| Ni2O2_id3361 | p4/mmm (LG61) | Ni@1a |  |
| Cr2S4_id441 | p4/mbm (LG63) | Cr@2a |  |
| Li2Mn2P2_id2976 | p4/nmm (LG64) | Mn@2a |  |
| Mn2Se2_id3049 | p4/nmm (LG64) | Mn@2a |  |
| Fe2Br2_id2236 | p4/nmm (LG64) | Fe@2a |  |
| Fe2Te2_id2298 | p4/nmm (LG64) | Fe@2a |  |
| Co2Li2Sb2_id2940 | p4/nmm (LG64) | Co@2a |  |
| Co2Na2Sb2_id917 | p4/nmm (LG64) | Co@2a |  |
| Co2S2_id1260 | p4/nmm (LG64) | Co@2a |  |
| KTiS2_id3097 | p3m1 (LG69) | Ti@1c |  |
| Ti2P2S6_id4036 | p-31m (LG71) | Ti@2b |  |
| V2Cl6_id3830 | p-31m (LG71) | V@2b | **QAH** |
| V2Br6_id2813 | p-31m (LG71) | V@2b | **QAH** |
| V2I6_id1801 | p-31m (LG71) | V@2b | **QAH** |
| Mn2Cl6_id1769 | p-31m (LG71) | Mn@2b | **QAH** |
| Fe2Cl6_id750 | p-31m (LG71) | Fe@2b | **QAH** |
| Fe2I6_id1760 | p-31m (LG71) | Fe@2b | **QAH** |
| Ru2Br6_id1788 | p-31m (LG71) | Ru@2b |  |
| Ru2I6_id2802 | p-31m (LG71) | Ru@2b | **QAH** |
| Sc2Cl2_id244 | p-3m1 (LG72) | Sc@2c |  |
| Sc2Br2_id223 | p-3m1 (LG72) | Sc@2c |  |
| Sc4N3_id2211 | p-3m1 (LG72) | Sc@2b, Sc@2c |  |
| Ti2N_id2541 | p-3m1 (LG72) | Ti@2c |  |
| Ti2Te2_id2408 | p-3m1 (LG72) | Ti@2c |  |
| Ti4C3_id186 | p-3m1 (LG72) | Ti@2b, Ti@2c |  |
| Cr2CS2_id2696 | p-3m1 (LG72) | Cr@2c |  |
| Cr2F2N_id688 | p-3m1 (LG72) | Cr@2c |  |
| Cr2S2_id1265 | p-3m1 (LG72) | Cr@2c |  |
| Cr4C3O2_id1188 | p-3m1 (LG72) | Cr@2b, Cr@2c |  |
| Cr4F2N3_id2198 | p-3m1 (LG72) | Cr@2b, Cr@2c |  |
| Cr4N3_id1197 | p-3m1 (LG72) | Cr@2b, Cr@2c |  |
| Cr4N3O2_id1191 | p-3m1 (LG72) | Cr@2b, Cr@2c |  |
| CrGa2Se4_id1054 | p-3m1 (LG72) | Cr@1a |  |
| Mn2CF2_id674 | p-3m1 (LG72) | Mn@2c |  |
| Mn2F2N_id2711 | p-3m1 (LG72) | Mn@2c |  |
| Mn2NO2_id691 | p-3m1 (LG72) | Mn@2c |  |
| Mn4C3F2_id173 | p-3m1 (LG72) | Mn@2b, Mn@2c |  |
| Mn4C3O2_id3218 | p-3m1 (LG72) | Mn@2b, Mn@2c |  |
| Mn4N3_id3227 | p-3m1 (LG72) | Mn@2b, Mn@2c |  |
| Mn4N3O2_id3221 | p-3m1 (LG72) | Mn@2b, Mn@2c |  |
| Fe2S2_id3313 | p-3m1 (LG72) | Fe@2c | **QAH** |
| Fe2Se2_id3314 | p-3m1 (LG72) | Fe@2c | **QAH** |
| Fe2Te2_id3315 | p-3m1 (LG72) | Fe@2c |  |
| FeO2_id456 | p-3m1 (LG72) | Fe@1a |  |
| Rh2F2_id2289 | p-3m1 (LG72) | Rh@2c |  |
| RhI2_id575 | p-3m1 (LG72) | Rh@2c |  |
| Y2Cl2_id30 | p-3m1 (LG72) | Y@2c |  |
| Y2I2_id2051 | p-3m1 (LG72) | Y@2c |  |
| Zr4C3_id187 | p-3m1 (LG72) | Zr@2b, Zr@2c |  |
| Cr3C2F2_id2170 | p-6m2 (LG78) | Cr@1a, Cr@2f |  |
| Cr3C2O2_id2173 | p-6m2 (LG78) | Cr@1a, Cr@2f |  |
| Mn3C2F2_id148 | p-6m2 (LG78) | Mn@1a, Mn@2f |  |
| Mn3C2O2_id151 | p-6m2 (LG78) | Mn@1a, Mn@2f |  |
| FeCl2_id2476 | p-6m2 (LG78) | Fe@1a |  |
| FeBr2_id2475 | p-6m2 (LG78) | Fe@1a |  |
| FeI2_id3494 | p-6m2 (LG78) | Fe@1a |  |
| Zr3N2_id2194 | p-6m2 (LG78) | Zr@1a, Zr@2f |  |
| Ti2Cl6_id1797 | p-62m (LG79) | Ti@2d |  |
| Zr2Cl6_id2819 | p-62m (LG79) | Zr@2d |  |
| Zr2Br6_id1806 | p-62m (LG79) | Zr@2d |  |
| Zr2I6_id1807 | p-62m (LG79) | Zr@2d |  |

# Supplementary Note 4. Results of monolayer hexagonal Fe_2_X_2_ (X = S, Se)

## I. Results of monolayer Fe_2_S_2_


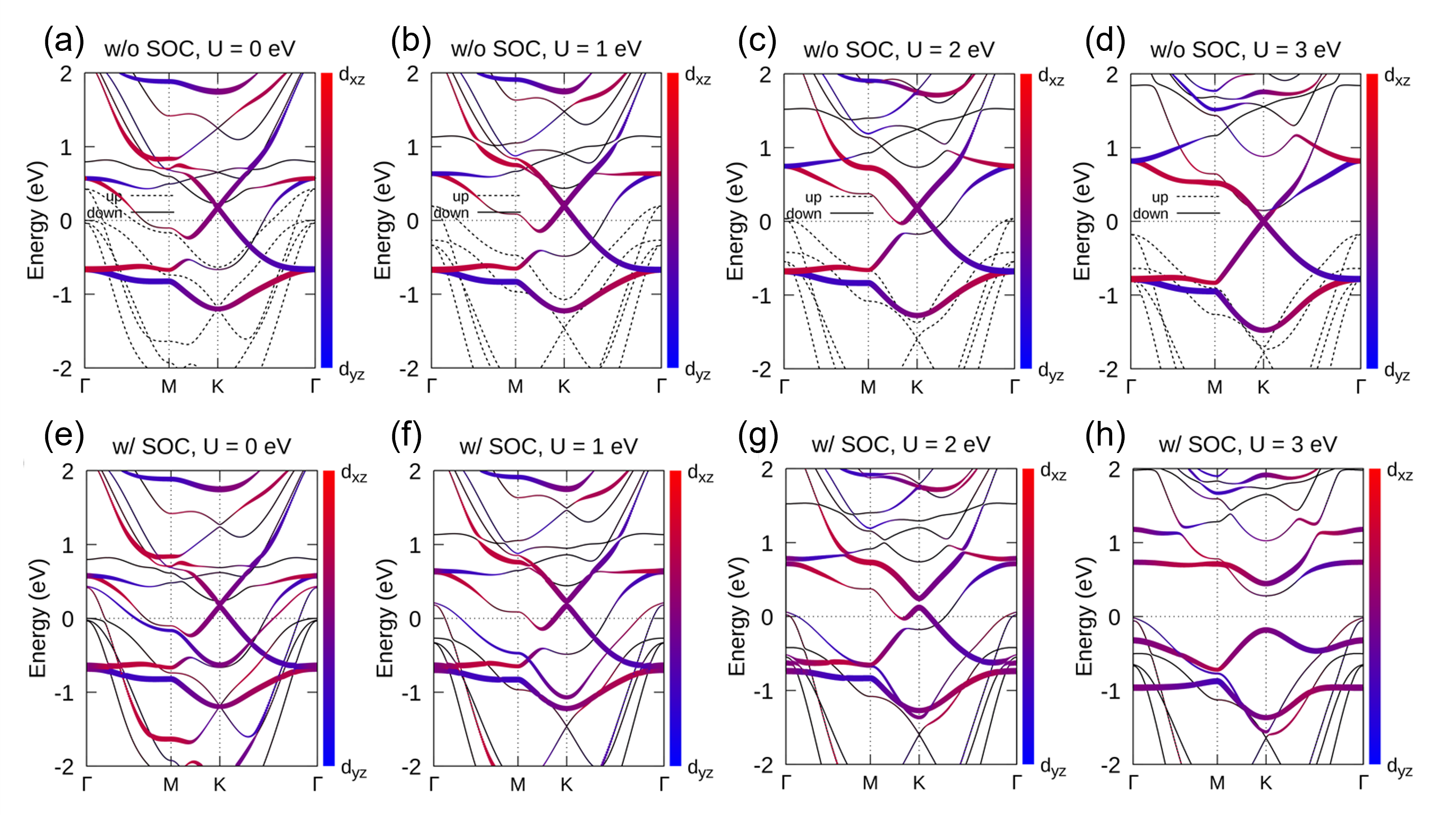


**Supplementary figure 1.** **Band structure of Fe_2_S_2_.** (a-d) Band structures of monolayer Fe_2_S_2_ without SOC calculated by the DFT+U method using different U values. (e-h) Band structures with SOC under different U values.


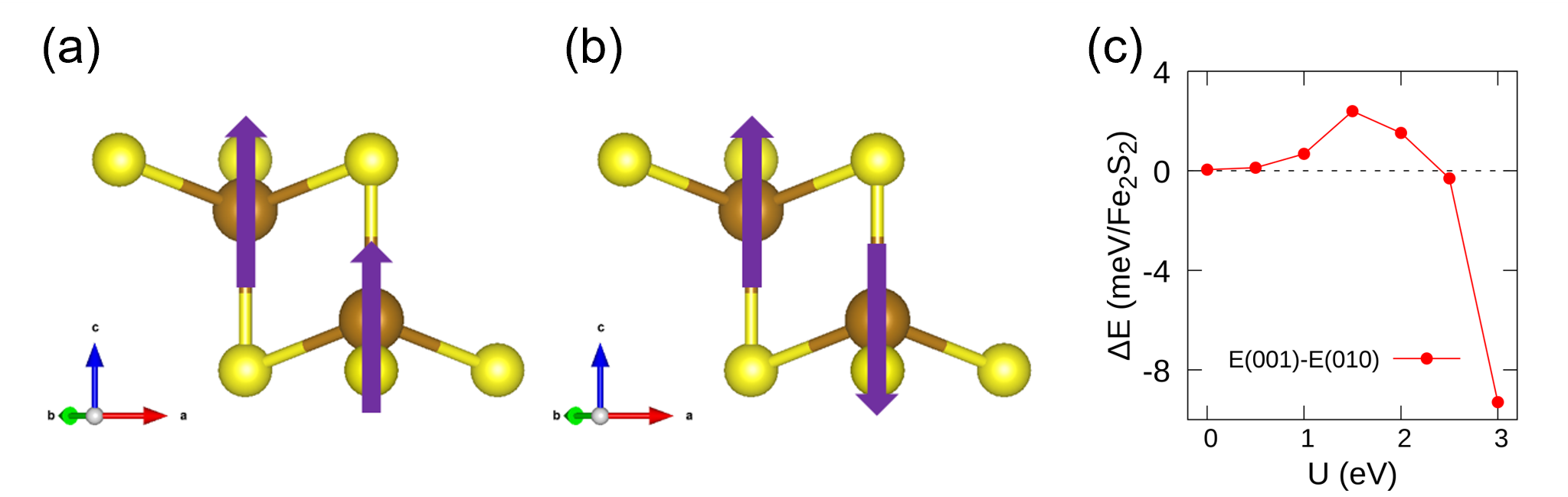


**Supplementary figure 2. Magnetic configurations and magnetocrystalline anisotropy energy.** (a-b) Monolayer Fe_2_S_2_ with (a) ferromagnetic (FM) and (b) antiferromagnetic (AFM) configurations. (c) Calculated magnetocrystalline anisotropy energy by the DFT+U method using different U values. FM state with magnetic moments parallel to *c*-axis is more stable than the AFM state by 227.5 meV/Fe_2_S_2_ (U = 3 eV).


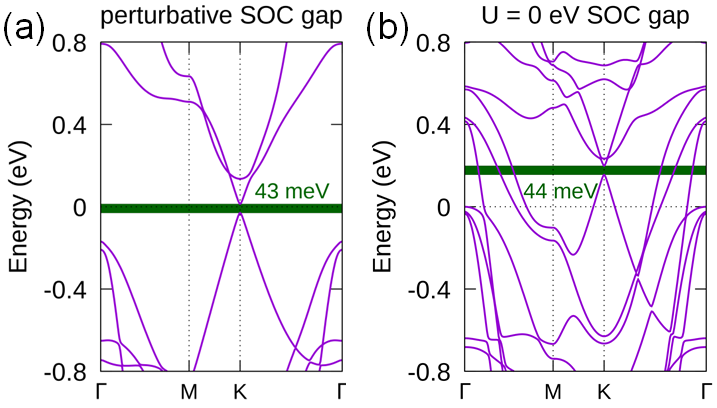


**Supplementary figure 3. Magnitude of the pure SOC gap.** (a) Pure SOC gap calculated by DFT+U method with U = 3 eV by treating SOC as a perturbation. (b) Pure SOC gap calculated by DFT+U method with U = 0 eV.


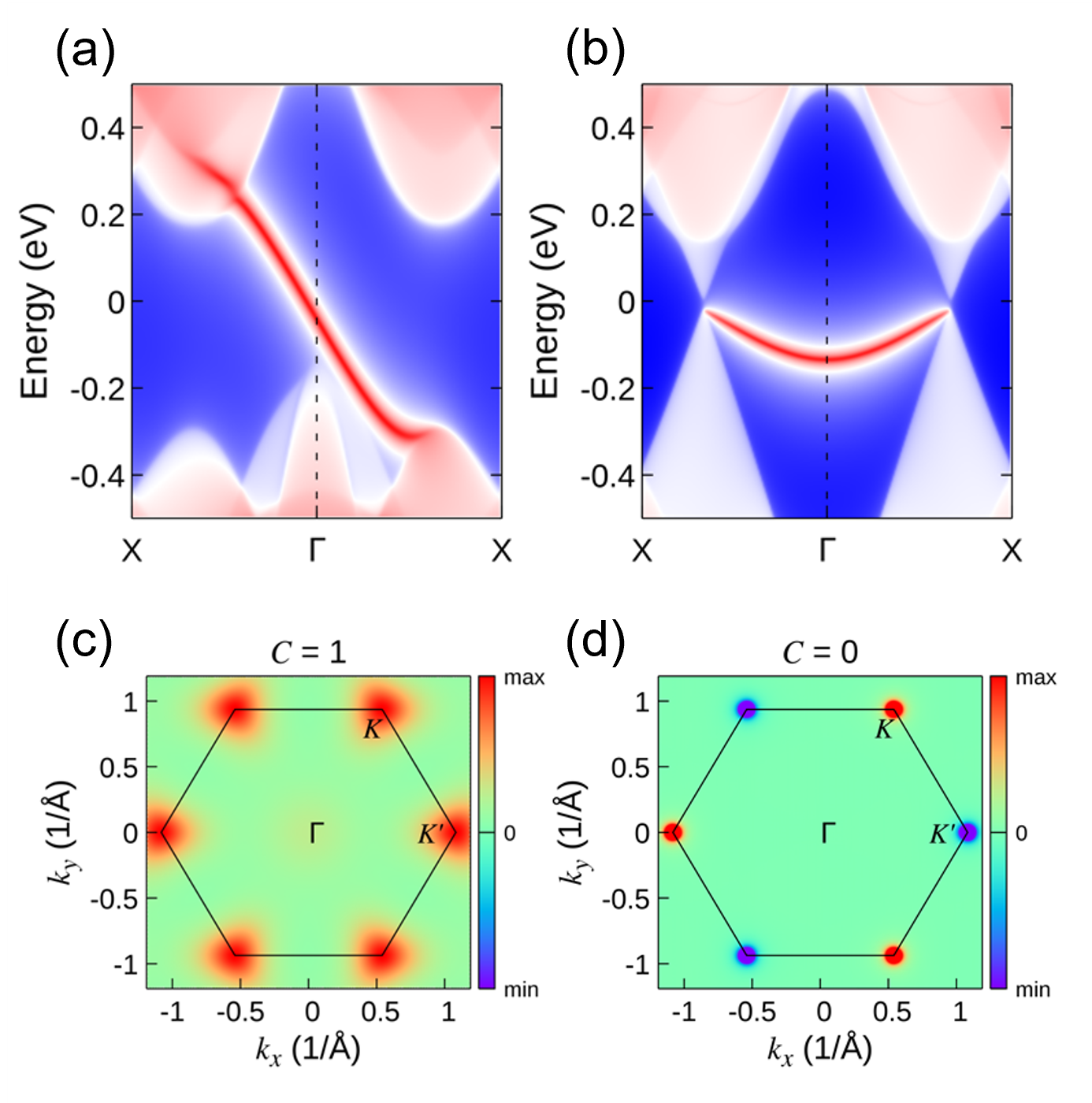


**Supplementary figure 4. Edge states and Berry curvature.** (a-b) Topological edge states calculated along the (100) direction (a) with SOC and (b) with 1 V/Å external electric field. The external electric field is parallel to the *c*-axis. (b) only shows the spin down channel. (c-d) Distribution of Berry curvature calculated (c) with SOC and (d) with 1 V/Å external electric field contributed by occupied valence bands in the momentum space. The external electric field is parallel to the *c*-axis. The hexagon represents the first Brillouin zone.


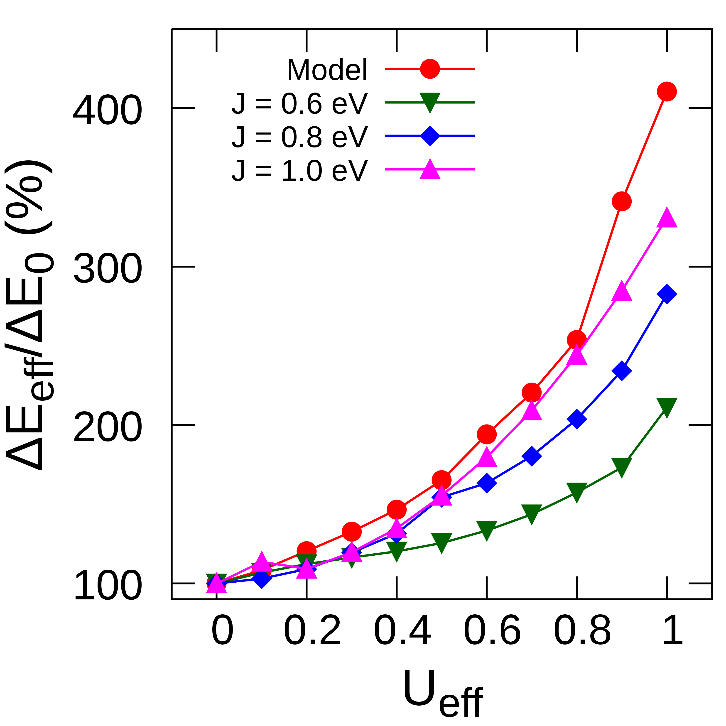


**Supplementary figure 5. SOC increment as a function of the effective parameter** $\boldsymbol{U}_{\mathbf{eff}}\boldsymbol{=U-3}\boldsymbol{J}$**.** The red curve is obtained by the four-band tight-binding model of Fe_2_S_2_ with interaction between parallel spin states considered only, while the other three curves are obtained by GGA+U calculations with different $U$ and $J$. Consistent tendency is shown from all results. As expected, the simplified correlation term is more accurate in a strong exchange case (large $J$), with a reasonable susceptibility $\chi/\hbar\approx0.73 \mathrm{eV}^{-1}$fitted by Eq. (11) [Eq. (4) in main text] with $J=1.0 \mathrm{eV}$.

## II. Results of monolayer Fe_2_Se_2_


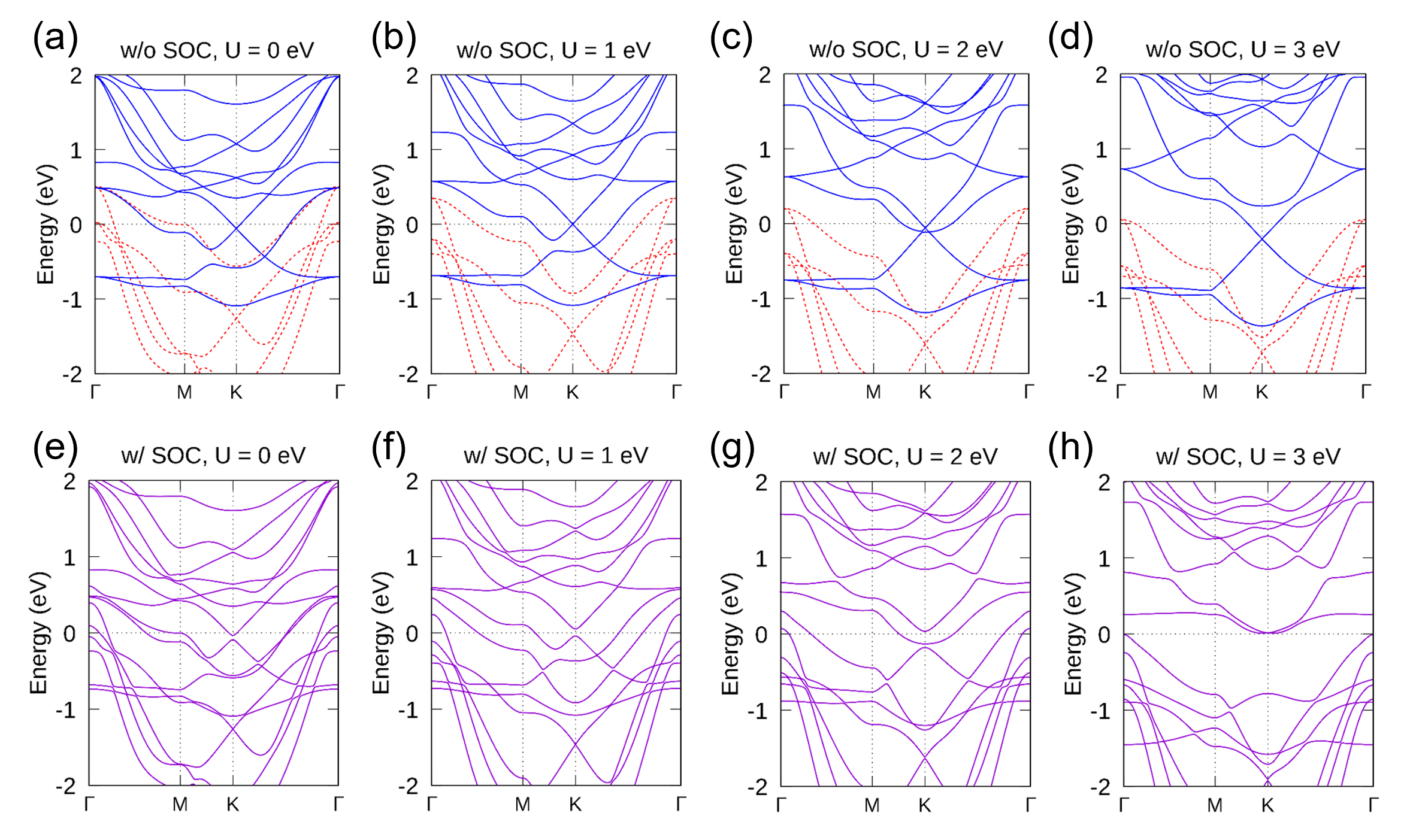


**Supplementary figure 6. Band structure of Fe_2_Se_2_.** (a-d) Band structures of monolayer Fe_2_Se_2_ without SOC calculated by the DFT+U method using different U values. (e-h) Band structures with SOC under different U values.


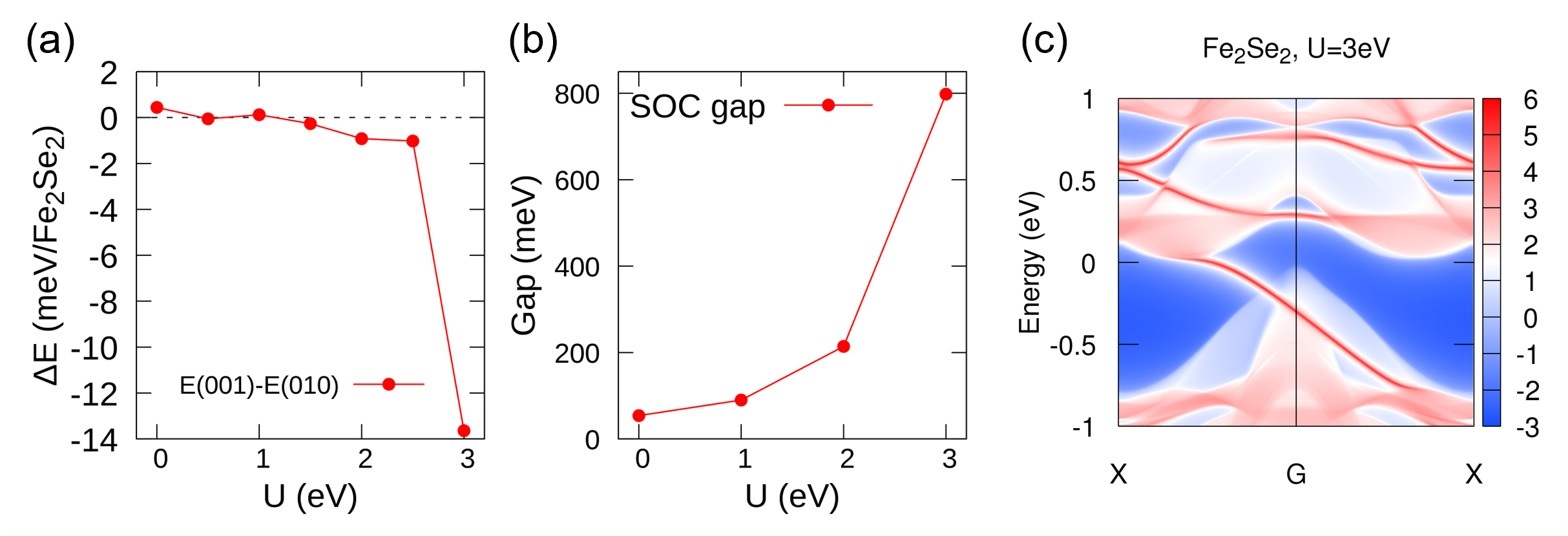


**Supplementary figure 7. Magnetocrystalline anisotropy energy, evolution of the nontrivial SOC gap, and topological edge states of Fe_2_Se_2_.** (a) Calculated magnetocrystalline anisotropy energy (MAE) by the DFT+U method using different U values. The FM state is more stable than the AFM state by 195.0 meV/Fe_2_Se_2_ (U = 3 eV). (b) Evolution of the nontrivial SOC gap of monolayer Fe_2_Se_2_ with the variation of U in DFT+U calculation. The SOC gap reaches 798 meV with U = 3 eV, while the system is a semiconductor with indirect band gap of 18 meV as shown in Fig. S5 (h). (c) Topological edge states calculated along the (100) direction with SOC, indicating the nonzero Chern number $C=1$.

# Supplementary Note 5. Spin-orbit gap enhanced by general correlation

In previous derivation, we concentrate on the strong spin-splitting limit that only interaction between electrons with parallel spins is considered. Here we perform a more general derivation of the enhanced SOC effect by counting all on-site correlation terms in the spin-splitting case. The general correlation Hamiltonian of $d$ orbitals is taken as the Kanamori Hamiltonian^3^:

$$\hat{H}_{C}^{K}=\frac{1}{2}\sum_{m\sigma} U\hat{n}_{m\sigma}\hat{n}_{m\bar{\sigma}}+\frac{1}{2}\sum_{m\neq m^{'},\sigma\sigma^{'}} \left( U^{'}-J\delta_{\sigma\sigma^{'}} \right)\hat{n}_{m\sigma}\hat{n}_{m^{'}\sigma^{'}} (14)$$

$$-\frac{1}{2}\sum_{m\neq m^{'}\sigma} J\hat{C}_{m\sigma}^{\dagger}\hat{C}_{m\bar{\sigma}}\hat{C}_{m^{'}\bar{\sigma}}^{\dagger}\hat{C}_{m^{'}\sigma}+\frac{1}{2}\sum_{m\neq m^{'}\sigma} J\hat{C}_{m\sigma}^{\dagger}\hat{C}_{m\bar{\sigma}}^{\dagger}\hat{C}_{m^{'}\bar{\sigma}}\hat{C}_{m^{'}\sigma},$$

where $U^{'}=U-2J$ for $d$ orbitals and $\bar{\sigma}=-\sigma$ denotes opposite spin. Four terms in Eq. (14) describe intra-orbital Coulomb repulsion, inter-orbital Coulomb repulsion and exchange attraction, spin flipping, and double hopping, sequentially. Here we take $E_{1}=\left\{ d_{xz},d_{yz} \right\}$ as example, the SOC Hamiltonian matrix written in bases $\left. |2,\pm1,\uparrow/\downarrow\right\rangle=-\left( \left. |d_{xz},\uparrow/\downarrow\right\rangle\pm i\left. |d_{yz},\uparrow/\downarrow\right\rangle\right)/\sqrt{2}$ was shown in Eq. (2):

$$\tilde{H}_{SOC}=\frac{\lambda\hbar^{2}}{2}\left( \begin{matrix} 1 & & & \\ & -1 & & \\ & & -1 & \\ & & & 1 \end{matrix} \right). (2)$$

In this base, the Kanamori Hamiltonian specifically reads

$$\hat{H}_{C}^{K}=U\left( \hat{n}_{+1,\uparrow}\hat{n}_{+1,\downarrow}+\hat{n}_{-1,\uparrow}\hat{n}_{-1,\downarrow} \right)+U^{'}\left( \hat{n}_{+1,\uparrow}\hat{n}_{-1,\downarrow}+\hat{n}_{+1,\downarrow}\hat{n}_{-1,\uparrow} \right) (15)$$

$$+\left（ U^{'}-J \right）\left( \hat{n}_{+1,\uparrow}\hat{n}_{-1,\uparrow}+\hat{n}_{+1,\downarrow}\hat{n}_{-1,\downarrow} \right)$$

$$-J\left( \hat{C}_{+1,\uparrow}^{\dagger}\hat{C}_{+1,\downarrow}\hat{C}_{-1,\downarrow}^{\dagger}\hat{C}_{-1,\uparrow}+\hat{C}_{+1,\downarrow}^{\dagger}\hat{C}_{+1,\uparrow}\hat{C}_{-1,\uparrow}^{\dagger}\hat{C}_{-1,\downarrow} \right)$$

$$+\frac{J}{2}\left( \hat{C}_{+1,\uparrow}^{\dagger}\hat{C}_{+1,\downarrow}^{\dagger}\hat{C}_{-1,\downarrow}\hat{C}_{-1,\uparrow}+\hat{C}_{+1,\downarrow}^{\dagger}\hat{C}_{+1,\uparrow}^{\dagger}\hat{C}_{-1,\uparrow}\hat{C}_{-1,\downarrow} \right)$$

$$+\frac{J}{2}\left( \hat{C}_{-1,\uparrow}^{\dagger}\hat{C}_{-1,\downarrow}^{\dagger}\hat{C}_{+1,\downarrow}\hat{C}_{+1,\uparrow}+\hat{C}_{-1,\downarrow}^{\dagger}\hat{C}_{-1,\uparrow}^{\dagger}\hat{C}_{+1,\uparrow}\hat{C}_{+1,\downarrow} \right),$$

where the double hopping term is written explicitly in a Hermitian form. Following the mean-field approach, we define $\bar{n}_{m\sigma}\equiv\left\langle\hat{n}_{m\sigma} \right\rangle$ and $\Phi_{m\sigma;m^{'}\sigma^{'}}\equiv\left\langle\hat{C}_{m\sigma}^{\dagger}\hat{C}_{m^{'}\sigma^{'}} \right\rangle$ as the expected values of the density and non-density term, respectively. Thus the mean-field level Kanamori Hamiltonian for doublet $E_{1}$ is

$$\tilde{H}_{c}^{K}\approx\left( \begin{matrix} \tilde{H}_{\uparrow} & \tilde{T} \\ \tilde{T}^{\dagger} & \tilde{H}_{\downarrow} \end{matrix} \right), (16)$$

where the Hamiltonian is written in block matrix form under the base $\tilde{C}=\left( \hat{C}_{+1,\uparrow},\hat{C}_{-1,\uparrow},\hat{C}_{+1,\downarrow},\hat{C}_{-1,\downarrow} \right)^{T}$ previously defined for SOC matrix Eq. (2), and the irrelevant constant term are omitted. Inside, $\tilde{H}_{\uparrow,\downarrow}$ denote the block that interaction between parallel spin states:

$$\tilde{H}_{\sigma}=$$

$$\left( \begin{matrix} U\bar{n}_{+1,\bar{\sigma}}+U^{'}\bar{n}_{-1,\bar{\sigma}}+\left( U^{'}-J \right)\bar{n}_{-1,\sigma} & J\mathrm{Re}\Phi_{+1,\bar{\sigma};-1,\bar{\sigma}} \\ J\mathrm{Re}\Phi_{+1,\bar{\sigma};-1,\bar{\sigma}} & U\bar{n}_{-1,\bar{\sigma}}+U^{'}\bar{n}_{+1,\bar{\sigma}}+\left( U^{'}-J \right)\bar{n}_{+1,\sigma} \end{matrix} \right),(17)$$

and $\tilde{T}$ contains spin flipping terms that connects each spin block:

$$\tilde{T}=-\frac{J}{2}\left( \begin{matrix} \Phi_{-1,\downarrow;-1,\uparrow} & \Phi_{+1,\downarrow;-1,\uparrow} \\ \Phi_{-1,\downarrow;+1,\uparrow} & \Phi_{+1,\downarrow;+1,\uparrow} \end{matrix} \right). (18)$$

As the SOC matrix is diagonal, the combination $\tilde{H}_{SOC}+\tilde{H}_{c}^{K}$ only modifies the diagonal block $\tilde{H}_{\sigma}$ into

$${\tilde{\mathcal{H}}}_{\sigma}=\tilde{H}_{\sigma}+\left( -1 \right)^{\sigma}\frac{\lambda\hbar^{2}}{2}\tau_{z}, (19)$$

with $\tau_{z}$ the Pauli matrix and $\left( -1 \right)^{\uparrow,\downarrow}\equiv\pm1$.

In a spin-splitting case, we concentrate on the spin-up channel. To obtain the effective Hamiltonian of the spin-up state, we introduce the Green’s function as $\hat{G}\left( \varepsilon\right)\equiv\left( \varepsilon-\tilde{H}_{SOC}-\tilde{H}_{c}^{K} \right)^{-1}$. Using the Dyson’s equation, we obtain the effective Green’s function of the spin-up block as $\tilde{G}_{\uparrow}^{-1}\left( \varepsilon\right)=\tilde{G}_{\uparrow,0}^{-1}\left( \varepsilon\right)-\tilde{\Sigma}_{\uparrow}\left( \varepsilon\right)$ where $\tilde{G}_{\uparrow,0}\left( \varepsilon\right)=\left( \varepsilon-{\tilde{\mathcal{H}}}_{\uparrow} \right)^{-1}$ is the bare Green’s function and $\tilde{\Sigma}_{\uparrow}\left( \varepsilon\right)=\tilde{T}\left( \varepsilon-{\tilde{\mathcal{H}}}_{\downarrow} \right)^{-1}\tilde{T}^{\dagger}$ is the self-energy. The effective Hamiltonian of the spin-up channel, given by $\tilde{G}_{\uparrow} \left( \varepsilon\right)=\left( \varepsilon-{\tilde{\mathcal{H}}}_{\uparrow}^{\mathrm{eff}} \right)^{-1}$, is then derived as

$${\tilde{\mathcal{H}}}_{\uparrow}^{\mathrm{eff}}={\tilde{\mathcal{H}}}_{\uparrow}+\tilde{T}\left( \varepsilon-{\tilde{\mathcal{H}}}_{\downarrow} \right)^{-1}\tilde{T}^{\dagger}. (20)$$

## I. Without spin-flipping, $\tilde{\boldsymbol{T}}\boldsymbol{=0}$

First, we consider the limit that spin-flipping is absent, i.e., $\tilde{T}=0$. In this limit, the effective Hamiltonian of spin-up state is identical to ${\tilde{\mathcal{H}}}_{\uparrow}$. Even so, the spin-down channel also matters the energy gap due to Coulomb repulsion ($U,U^{'}$). To see it, we decompose ${\tilde{\mathcal{H}}}_{\uparrow}$ of Eq. (17) into two parts since $\left( \begin{matrix} A & 0 \\ 0 & B \end{matrix} \right)=\frac{A+B}{2}+\frac{A-B}{2}\tau_{z}$. Therefore, we have

$${\tilde{\mathcal{H}}}_{\uparrow}=\frac{\left( U+U^{'} \right)\bar{n}_{\downarrow}+\left( U^{'}-J \right)\bar{n}_{\uparrow}}{2} (21)$$

$$+\left( \begin{matrix} \frac{2J\bar{L}_{z,\downarrow}-\left( U^{'}-J \right)\bar{L}_{z,\uparrow}+\lambda\hbar^{3}}{2\hbar} & J\mathrm{Re}\Phi_{+1,\downarrow;-1,\downarrow} \\ J\mathrm{Re}\Phi_{+1,\downarrow;-1,\downarrow} & -\frac{2J\bar{L}_{z,\downarrow}-\left( U^{'}-J \right)\bar{L}_{z,\uparrow}+\lambda\hbar^{3}}{2\hbar} \end{matrix} \right),$$

where the conventions $\bar{n}_{\sigma}=\bar{n}_{+1,\sigma}+\bar{n}_{-1,\sigma}$ and $\bar{L}_{z,\sigma}=\bar{n}_{+1,\sigma}+\bar{n}_{-1,\sigma}$ are used. Two energies are solved as $E_{\uparrow,\pm}=\frac{2\left( U-J \right)\bar{n}_{\downarrow}+\left( U-3J \right)\bar{n}_{\uparrow}}{2}\pm\Delta E_{\uparrow}/2$, with a gap $\Delta E_{\uparrow}=E_{\uparrow,+}-E_{\uparrow,-}$ as

$$\Delta E_{\uparrow}=2\sqrt{\frac{1}{4\hbar^{2}}\left[ 2J\bar{L}_{z,\downarrow}-\left( U-3J \right)\bar{L}_{z,\uparrow}+\lambda\hbar^{3} \right]^{2}+J^{2}\phi_{0}^{2}}. (22)$$

where $\phi_{o}\equiv\mathrm{Re}\Phi_{+1,\downarrow;-1,\downarrow}$ denotes the orbital flipping effect. Expanding $\Delta E_{\uparrow}$ up to the linear order of $\lambda$, we obtain the effective energy gap as

$$\Delta E_{\uparrow}\approx\Delta E_{0}+\frac{2J\left| \bar{L}_{z,\downarrow} \right|+\left( U-3J \right)\left| \bar{L}_{z,\uparrow} \right|}{\hbar}, (23)$$

with $\left| \bar{L}_{z,\downarrow} \right|$ assumed as the linear function of $\lambda$, and $\Delta E_{0}=\lambda\hbar^{2}$ used. Eq. (23) is the modified result of Eq. (10) [Eq. (3) in the main text] with further considering the first order effect of the opposite spin state. Namely, the orbital polarization of the opposite spin states increases the energy gap as $\left| \bar{L}_{z,\uparrow} \right|$ does. In the strong splitting limit that spin-down state is fully occupied or unoccupied, i.e., $\bar{L}_{z,\downarrow}=0$, Eq. (23) recovers the previous result Eq. (10).

**II. With spin-flipping,** $\tilde{\boldsymbol{T}}\boldsymbol{\neq0}$

Before evaluating the effective Hamiltonian in Eq. (20), we assume that spin flipping terms between the same orbital are orbital-independent $\Phi_{\pm1,\downarrow;\pm1,\uparrow}\equiv\phi_{s}$ and dominates the spin-orbit flipping terms $\Phi_{\pm1,\downarrow;\mp1,\uparrow}\approx0$. The self-energy is then approximated as

$$\tilde{\Sigma}_{\uparrow}\left( \varepsilon\right)\approx\frac{J^{2}\left| \phi_{s} \right|^{2}}{4}\frac{1}{\left( \varepsilon-E_{\downarrow,+} \right)\left( \varepsilon-E_{\downarrow,-} \right)} (24)$$

$$\times\left( \begin{matrix} \varepsilon-U\bar{n}_{-1,\uparrow}-U^{'}\bar{n}_{+1,\uparrow}-\left( U^{'}-J \right)\bar{n}_{+1,\downarrow}-\lambda& -J\phi_{o} \\ -J\phi_{o} & \varepsilon-U\bar{n}_{+1,\uparrow}-U^{'}\bar{n}_{-1,\uparrow}-\left( U^{'}-J \right)\bar{n}_{-1,\downarrow} \end{matrix} \right),$$

where $E_{\downarrow,\pm}$ is the energy level of the spin-down channel

$$E_{\downarrow\pm}=\frac{\left( U+U^{'} \right)\bar{n}_{\uparrow}+\left( U^{'}-J \right)\bar{n}_{\downarrow}}{2} (25)$$

$$\pm\sqrt{\frac{1}{4\hbar^{2}}\left[ 2J\bar{L}_{z,\uparrow}-\left( U'-J \right)\bar{L}_{z,\downarrow}+\lambda\hbar^{3} \right]^{2}+J^{2}\phi_{0}^{2}}.$$

Therefore, the effective Hamiltonian of the spin-up state Eq. (20) is

$${\tilde{\mathcal{H}}}_{\uparrow}^{\mathrm{eff}}\approx\left( \begin{matrix} \frac{2J\bar{L}_{z,\downarrow}-\left( U^{'}-J \right)\bar{L}_{z,\uparrow}+\lambda\hbar^{3}}{2\hbar} & J\phi_{o} \\ J\phi_{o} & -\frac{2J\bar{L}_{z,\downarrow}-\left( U^{'}-J \right)\bar{L}_{z,\uparrow}+\lambda\hbar^{3}}{2\hbar} \end{matrix} \right) (26)$$

$$+\frac{J^{2}\left| \phi_{s} \right|^{2}}{4}\frac{1}{\left( \varepsilon-E_{\downarrow,+} \right)\left( \varepsilon-E_{\downarrow,-} \right)}\left( \begin{matrix} \frac{2J\bar{L}_{z,\uparrow}-\left( U^{'}-J \right)\bar{L}_{z,\downarrow}-\lambda\hbar^{3}}{2\hbar} & -J\phi_{o} \\ -J\phi_{o} & -\frac{2J\bar{L}_{z,\uparrow}-\left( U^{'}-J \right)\bar{L}_{z,\downarrow}-\lambda\hbar^{3}}{2\hbar} \end{matrix} \right),$$

where the terms with identity matrix are omitted. Taking $\varepsilon$ as the mean value of the spin-up channel, i.e., $\varepsilon\approx\frac{\left( E_{\uparrow+}+E_{\uparrow-} \right)}{2}=\frac{\left( U+U^{'} \right)\bar{n}_{\downarrow}+\left( U^{'}-J \right)\bar{n}_{\uparrow}}{2}$, the energy-resolved factor becomes

$$\left( \varepsilon-E_{\downarrow,+} \right)\left( \varepsilon-E_{\downarrow,-} \right)=\frac{\left( U+J \right)^{2}\left( \bar{n}_{\downarrow}-\bar{n}_{\uparrow} \right)}{4}-\left( \Delta E_{\downarrow} \right)^{2}\approx\frac{\left( U+J \right)^{2}\left( \bar{n}_{\downarrow}-\bar{n}_{\uparrow} \right)}{4}. (27)$$

Hence, ${\tilde{\mathcal{H}}}_{\uparrow}^{\mathrm{eff}}$ now is

$${\tilde{\mathcal{H}}}_{\uparrow}^{\mathrm{eff}}=\frac{2J\left( \bar{L}_{z,\downarrow}+\alpha\bar{L}_{z,\uparrow} \right)-\left( U^{'}-J \right)\left( \bar{L}_{z,\uparrow}+\alpha\bar{L}_{z,\downarrow} \right)+\left( 1-\alpha\right)\lambda\hbar^{3}}{2\hbar}\tau_{z} (28)$$

$$+\left( 1-\alpha\right)J\phi_{o}\tau_{x},$$

with $\alpha=\frac{J^{2}}{\left( U+J \right)^{2}}\left| \frac{\phi_{s}}{\bar{n}_{\downarrow}-\bar{n}_{\uparrow}} \right|^{2}$. Therefore, we obtain a more general effective gap as

$$\Delta E_{\uparrow}\approx\Delta E_{\mathrm{eff}}+\frac{2J}{\hbar}\left| \bar{L}_{z,\downarrow} \right|-\left| \frac{\phi_{s}}{\bar{n}_{\downarrow}-\bar{n}_{\uparrow}} \right|^{2}\Gamma\left( \bar{L}_{z,\uparrow},\bar{L}_{z,\downarrow} \right), (29)$$

where

$$\Gamma\left( \bar{L}_{z,\uparrow},\bar{L}_{z,\downarrow} \right)=\frac{J^{2}}{\left( U+J \right)^{2}}\left( \lambda\hbar^{2}+\frac{2J\left| \bar{L}_{z,\uparrow} \right|+\left( U-3J \right)\left| \bar{L}_{z,\downarrow} \right|}{\hbar} \right), (30)$$

and $\Delta E_{\mathrm{eff}}$ is defined in Eq. (10) [Eq. (3) in main text]. Besides the previously obtained gap $\Delta E_{\mathrm{eff}}$, the orbital polarization of the opposite spin channel $\bar{L}_{z, \bar{\sigma}}$ and a spin-flipping-induced term $\propto\phi_{s}^{2}=\left\langle\hat{C}_{m\sigma}^{\dagger}\hat{C}_{m\bar{\sigma}} \right\rangle^{2}$ appear, where $\bar{n}_{\sigma}-\bar{n}_{\bar{\sigma}}$ counts the exchange effect. With zero spin flipping, the energy gap is still enhanced even the opposite spin channel is considered. On the other hand, finite spin flipping $\phi_{s}\neq0$ will reduce the enhancement, as the flipping weakens the orbital polarization of each channel. Nevertheless, a relative strong exchange splitting $\left| \bar{n}_{\sigma}-\bar{n}_{\bar{\sigma}} \right|\gg\left| \phi_{s} \right|$ in 3*d* systems renders our theory and design principles approximately valid.

# Supplementary Note 6. Exchange coupling and magnetocrystalline anisotropy energy

For an arbitrarily oriented spin, the SOC Hamiltonian reads^4, 5^

$$H_{SOC}=\left[ \frac{1}{2}L_{+}e^{-i\phi}\left( \cos\theta S_{x^{'}}-iS_{y^{'}}+\sin\theta S_{z^{'}} \right) \right. (31)$$

$$+\frac{1}{2}L_{-}e^{i\phi}\left( \cos\theta S_{x^{'}}+iS_{y^{'}}+\sin\theta S_{z^{'}} \right)$$

$$+\left. L_{z}\left( -\sin\theta S_{x^{'}}+\cos\theta S_{z^{'}} \right) \right],$$

where $\mathbf{n}=\left( \sin\theta\cos\phi,\sin\theta\sin\phi,\cos\theta\right)$ is the direction of the spin. In the strong exchange limit in 3*d* systems, electrons near the Fermi surface are spin polarized, and SOC effect is dominated by the spin-conserved part

$$H_{SOC}^{sc}=\lambda S_{z^{'}}\left( L_{z}\cos\theta+\frac{1}{2}L_{+}e^{-i\phi}\sin\theta+\frac{1}{2}L_{-}e^{i\phi}\sin\theta S_{z^{'}} \right). (32)$$

For distinct frontier orbital, the SOC splitting energy result in the magnetic anisotropy.

## I. Orbital doublet $\boldsymbol{E}_{\boldsymbol{1}}\boldsymbol{=}\left\{ \boldsymbol{d}_{\boldsymbol{xz}}\boldsymbol{,}\boldsymbol{d}_{\boldsymbol{yz}} \right\}$

Considering single spin channel of $\left\{ \left. |d_{xz};\mathbf{n},\uparrow\right\rangle,\left. |d_{yz};\mathbf{n},\uparrow\right\rangle\right\}$ under half-filling, the spin-conserved SOC Hamiltonian reads

$$\tilde{H}_{SOC}^{sc}=\frac{\lambda\hbar^{2}}{2}\left( \begin{matrix} 0 & -i\cos\theta\\ i\cos\theta& 0 \end{matrix} \right)\underset{\to}{diag.}\left( \begin{matrix} -\frac{\lambda\hbar^{2}}{2}\cos\theta& 0 \\ 0 & \frac{\lambda\hbar^{2}}{2}\cos\theta\end{matrix} \right), (33)$$

where $diag.$ denotes diagonalization of the matrix. Then the orbital degeneracy is splitted, and the occupied state takes the energy of $E=-\frac{\lambda\hbar^{2}}{2}\left| \cos\theta\right|$, which is minimized when $\theta=0$ or $\pi$, making out-of-plane magnetization favored.

## II. Orbital doublet $\boldsymbol{E}_{\boldsymbol{2}}\boldsymbol{=}\left\{ \boldsymbol{d}_{\boldsymbol{xy}}\boldsymbol{,}\boldsymbol{d}_{\boldsymbol{x}^{\boldsymbol{2}}\boldsymbol{-}\boldsymbol{y}^{\boldsymbol{2}}} \right\}$

In the subspace of $\left\{ \left. |d_{x^{2}-y^{2}};\mathbf{n},\uparrow\right\rangle,\left. |d_{xy};\mathbf{n},\uparrow\right\rangle\right\}$, the spin-conserved SOC Hamiltonian reads

$$\tilde{H}_{SOC}^{sc}=\frac{\lambda\hbar^{2}}{2}\left( \begin{matrix} 0 & 2i\cos\theta\\ -2i\cos\theta& 0 \end{matrix} \right)\underset{\to}{diag.}\left( \begin{matrix} -\lambda\hbar^{2}\cos\theta& 0 \\ 0 & \lambda\hbar^{2}\cos\theta\end{matrix} \right), (34)$$

where the occupied energy $E=-\lambda\hbar^{2}\left| \cos\theta\right|$ is also minimized when $\theta=0$ or $\pi$.

## III. Orbital triplet $\boldsymbol{T=}\left\{ \boldsymbol{d}_{\boldsymbol{xy}}\boldsymbol{,}\boldsymbol{d}_{\boldsymbol{yz}}\boldsymbol{,}\boldsymbol{d}_{\boldsymbol{xz}} \right\}$

In the subspace of $\left\{ \left. |d_{xy};\mathbf{n},\uparrow\right\rangle,\left. |d_{yz};\mathbf{n},\uparrow\right\rangle,\left. |d_{xz};\mathbf{n},\uparrow\right\rangle\right\}$, the spin-conserved SOC Hamiltonian becomes

$$\tilde{H}_{SOC}^{sc}=\frac{\lambda\hbar^{2}}{2}\left( -\begin{matrix} 0 & i\sin\theta\sin\phi& -i\sin\theta\cos\phi\\ i\sin\theta\sin\phi& 0 & i\cos\theta\\ i\sin\theta\cos\phi& -i\cos\theta& 0 \end{matrix} \right) (35)$$

$$\underset{\to}{diag.}\left( \begin{matrix} -\frac{\lambda\hbar^{2}}{2} & 0 & 0 \\ 0 & 0 & 0 \\ 0 & 0 & \frac{\lambda\hbar^{2}}{2} \end{matrix} \right).$$

The perturbed energies are independent to the orientation of the magnetization, indicating that the first order SOC effect is not the origin of the magnetic anisotropy for the orbital triplet in 3D lattice.

Therefore, for orbital doublets $E_{1}=\left\{ d_{xz},d_{yz} \right\}$ and $E_{2}=\left\{ d_{xy},d_{x^{2}-y^{2}} \right\}$, the out-of-plane magnetization is favored with the energy of the magnetic anisotropy proportional to the SOC parameter $\lambda$. With the correlation effect combined, the SOC effect is enhanced via the augment $\lambda\to\lambda_{\mathrm{eff}}$, resulting in a significant gain of the magnetic anisotropy energy. Such an enhancement is efficient especially in the 2D case to stabilize the long-range magnetic order normal to the 2D plane.

**Supplementary references**

1. Haastrup S.*, et al.* The Computational 2D Materials Database: high-throughput modeling and discovery of atomically thin crystals. *2D Materials* **5**, 042002 (2018).

2. Gjerding M. N.*, et al.* Recent progress of the computational 2D materials database (C2DB). *2D Materials* **8**, 044002 (2021).

3. Kanamori J. Electron Correlation and Ferromagnetism of Transition Metals. *Prog. Theor. Phys.* **30**, 275-289 (1963).

4. Dai D., Xiang H., Whangbo M.-H. Effects of spin-orbit coupling on magnetic properties of discrete and extended magnetic systems. *J. Comput. Chem.* **29**, 2187-2209 (2008).

5. Wang D., Tang F., Du Y., Wan X. First-principles study of the giant magnetic anisotropy energy in bulk Na_4_IrO_4_. *Phys. Rev. B* **96**, 205159 (2017).
